# Genome Sequence of a Spodoptera frugiperda Multiple Nucleopolyhedrovirus Isolated from Fall Armyworm (Spodoptera frugiperda) in Nigeria, West Africa

**DOI:** 10.1128/MRA.00565-21

**Published:** 2021-08-26

**Authors:** Jörg T. Wennmann, Ghislain T. Tepa-Yotto, Johannes A. Jehle, Georg Goergen

**Affiliations:** a Julius Kühn Institute (JKI)-Federal Research Centre for Cultivated Plants, Institute for Biological Control, Darmstadt, Germany; b Biorisk Management Facility, International Institute of Tropical Agriculture, Cotonou, Benin; c Ecole de Gestion et de Production Végétale et Semencière, Université Nationale d’Agriculture, Kétou, Benin; KU Leuven

## Abstract

We report the entire genome sequence of an isolate of Spodoptera frugiperda multiple nucleopolyhedrovirus from Nigeria, West Africa. The genome is 132,710 bp long and contains 144 open reading frames. The GC content is 40.3% and, based on baculovirus species demarcation criteria, the isolate belongs to the species Spodoptera frugiperda *multiple nucleopolyhedrovirus*.

## ANNOUNCEMENT

The fall armyworm (FAW) (Spodoptera frugiperda [J. E. Smith] [Lepidoptera, Noctuidae]) is native to the Americas and is a serious pest in maize and other crops (1). The FAW was introduced from South America to the African continent, where it has rapidly spread throughout sub-Saharan Africa and further to Yemen, India, and other Asian countries (2–4). In its new habitats, naturally occurring and indigenous antagonists of FAW are being sought to be used as biological control agents. An option to manage FAW is the use of Spodoptera frugiperda multiple nucleopolyhedrovirus (SfMNPV) (family *Baculoviridae*, genus *Alphabaculovirus*), commercial formulations of which are successfully used to control FAW in the Americas.

A new SfMNPV isolate from Nigeria (SfMNPV-KA1) was obtained from two infected second- and fourth-instar FAW larvae that were collected in the Sudan-Guinea savannah on 9 September 2017 from a maize field in Checheyi village (Kwali-Abuja, Nigeria [8°57′36″N, 7°29′13″E]) (collected by G. Tepa-Yotto). The sampled maize field was planted on 8 August 2017 and remained unsprayed until the collection date. The sample was transferred to the International Institute of Tropical Agriculture (IITA)-Benin for laboratory multiplication (import permit 130/DIP-09/2017/SPVCP and quarantine inspection report and multiplication authorization 0029/IP-11/2017/SPVCP/CQF-A) and for subsequent identification at Julius Kühn Institute (JKI) (Darmstadt, Germany). For virus occlusion body (OB) isolation, the larval cadavers were homogenized with a micropestle in 1 ml of 0.5% SDS, followed by sucrose gradient ultracentrifugation (5). The DNA was purified following phenol-chloroform extraction and isoamyl alcohol precipitation (5). SfMNPV was identified by PCR using degenerate oligonucleotide primers targeting the *polyhedrin* (*polh*) (prPH-1, 5′-M13-NRCNGARGAYCCNTT-3′; prPH-2, 5′-M13-DGGNGCRAAYTCYTT-3′), *late expression factor 8* (*lef-8*) (prL8-1, 5′-M13-CAYGGHGARATGAC-3′; prL8-2, 5′-M13-AYRTASGGRTCYTCSGC-3′), and *late expression factor 9* (*lef-9*) (prL9-1, 5′-M13-AARAAYGGITAYGCBG-3′; prL9-2, 5′-M13-TTGTCDCCRTCRCARTC-3′) baculovirus genes (6). The partial *polh*, *lef-8*, and *lef-9* fragments were Sanger sequenced using the same primer pairs (6). BLAST searches of the partial gene sequences confirmed SfMNPV within the sample. The virus isolate was termed SfMNPV-KA1, and its whole genome was sent for sequencing (StarSeq GmbH, Mainz, Germany) (Illumina NextSeq 500 sequencer, NEBNext Ultra II DNA library preparation kit, and 5 million paired-end reads 150 nucleotides [nt] in length). The reads were quality filtered and adapter trimmed using Trim Galore v0.6.3 (7), with quality (Phred) scores of >30 and a minimal read length of 51 nt. The quality-filtered reads were used to assemble *de novo* the 132,710-bp-long SfMNPV-KA1 genome (CLC *de novo* assembly tool v1.0.0 [with default parameters]; Qiagen GmbH, Hilden, Germany). Quality filtering and genome assembly were conducted on a Galaxy server of the JKI. The genome was circularized by overlapping ends using Geneious Prime v2021.0.1 (Biomatters Ltd., Auckland, New Zealand), and the *polh* gene was set as the first open reading frame (ORF) in the genome. In total, 144 ORFs (at least 150 nt long, with overlap shorter than 50 nt) were found using the annotation tool in Geneious Prime. The GC content was 40.3%. The average read depth was 4,881× ± 398×. To check for any intraisolate genetic variation, a bacsnp analysis (8) for single-nucleotide polymorphisms (SNPs) was performed, but it did not find any SNP-based variation within the SfMNPV-KA1 sample, indicating that the isolate appeared to be extremely homogeneous. The entire genome of SfMNPV-KA1 was aligned with previously sequenced SfMNPV isolates from the Americas using the Mauve genome aligner (with default settings) as implemented in Geneious Prime. The alignment included isolates from the United States (SfMNPV-3AP2 [GenBank accession number EF035042]), Brazil (SfMNPV-19 [GenBank accession number EU258200]), Colombia (SfMNPV-Colombia [GenBank accession number KF891883]), and Nicaragua (SfMNPV-NIC [GenBank accession number HM595733] and SfMNPV-G defective [GenBank accession number JF899325]), the genomes of which range between 128,034 bp (SfMNPV-G defective) and 134,239 bp (SfMNPV-Colombia) (9, 10). According to the demarcation criteria for baculovirus species that are based on the Kimura two-parameter distances of *polh/lef-8/lef-9* partial genes or the 38 baculovirus core genes found in all fully sequenced baculovirus genomes (6, 11), SfMNPV-KA1 was confirmed to belong to the Spodoptera frugiperda *multiple nucleopolyhedrovirus* species, with SfMNPV-19 (132,565 bp, with 141 ORFs) from Brazil (12) being the most closely related taxon (pairwise identity of 99.8%). The close relationship of SfMNPV-KA1 to SfMNPV-19 makes the Americas as its natural origin most likely and suggests that SfMNPV might have accompanied FAW to newly invaded habitats in Africa. The hypothesis that SfMNPV-KA1 is derived from experimental sprays of commercial SfMNPV formulations can be excluded, because such trials started only in 2019 and in other states within Nigeria.

### Data availability.

The sequence reads are available at the NCBI Sequence Read Archive (SRA) under the BioProject number PRJNA731917. The *de novo* assembled SfMNPV-KA1 consensus sequence was deposited in GenBank under the accession number MZ292981.
